# Utilizing Engineered Monobodies for the Electrochemical Quantification of Lysozyme

**DOI:** 10.1021/acs.analchem.5c03304

**Published:** 2025-12-14

**Authors:** Sunanda Dey, Andrew Naser, Daniel R. Woldring, David P. Hickey

**Affiliations:** Department of Chemical Engineering and Materials Science, 3078 Michigan State University, East Lansing, Michigan 48824, United States

## Abstract

Monobody binding proteins are derived from the Fn3 domain of human fibronectin. These robust proteins can be engineered to bind to a wide range of small molecule-, nucleotide-, or large biomolecule-based analytes with high specificity and stability, thus making them ideal for biosensing applications. Here, we demonstrate an electrochemical biosensor utilizing an engineered monobody as a biorecognition element for the detection of lysozyme as a model biomarker. An engineered monobody binding protein was immobilized onto glassy carbon electrodes through a process of electrochemical grafting to create the sensing interface, while a water-soluble ferrocene derivative was used as an electrochemical indicator. Square wave voltammetry of resulting monobody-modified electrodes revealed a significant decrease in peak current density upon incubation with 24 μM lysozyme (250 ± 20 μA cm^–2^ decrease in peak current compared to 20 ± 9 μA cm^–2^ decrease upon incubation with 24 μM bovine serum albumin as a negative control), and the sensor exhibited a linear detection range up to 1 μM lysozyme (with a sensitivity of 129 μA cm^–2^ μM^–1^, a limit of quantification of 290 nM and a limit of detection of 87 nM). Measurements taken from lysozyme samples in diluted canine serum indicate that the sensor maintains high specificity and sensitivity in a complex biological medium with small amounts of nonspecific adsorption. This work demonstrates significant potential for monobodies to expand the existing toolkit of electrochemical biorecognition elements while enhancing the performance and reliability of portable diagnostic devices.

## Graphical abstract

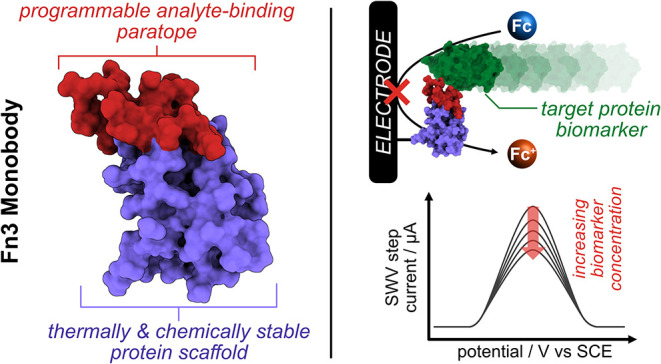

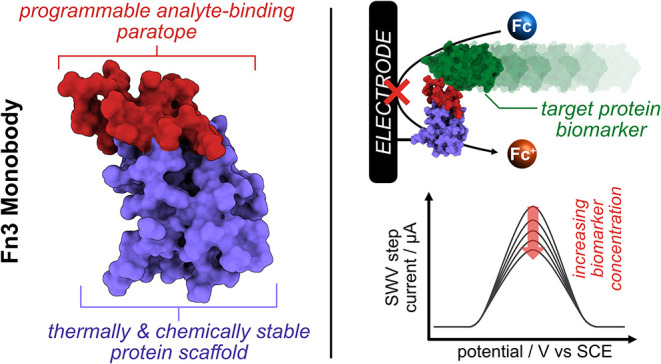

## Introduction

Electrochemical biosensors have emerged as effective tools for the rapid and specific detection of various analytes across biomedical research, clinical diagnostics, and environmental monitoring.^1–6^ These biosensors utilize a variety of biorecognition elements – such as enzymes, antibodies, and aptamers – to accurately identify and quantify target analytes with exceptionally high specificity.^7–13^ Despite the success of these recognition elements in a variety of applications, detecting larger biomolecules like nucleic acids and proteins using electrochemical biosensors remains challenging due to nonspecific binding and the limited availability of suitable diagnostic enzyme/analyte pairs.^14,15^ Recently, monobodies have been explored as robust and modular biorecognition elements in nonelectrochemical applications.^16^ Monobodies (Mbs) are a class of “antibody mimetic” proteins that are highly robust and can be tailored to bind to a wide range of small molecule, protein, and nucleic acid–based analytes with high specificity. Unlike antibodies, Mbs are small synthetic proteins (∼10 kDa compared to ∼150 kDa for antibodies) that are highly modular and can be manufacturer in bacteria with high yields for commercial applications.^17^ Consequently, we considered the possibility of employing monobodies as biorecognition elements in an electrochemical biosensor to combine the modularity and reproducibility of aptameric biosensors with the thermal and chemical stability of enzyme- and antibody-based electrochemical biosensors.

Monobodies are synthetic binding proteins derived from a human fibronectin type 3 (Fn3) domain as a robust molecular scaffold.^17^ The core Fn3 scaffold consists of seven β-sheets (labeled A-G) that form a thermally stable immunoglobulin fold; this tertiary structure combined with a lack of disulfide bonds enables the Fn3 scaffold to reliably refold even after thermal denaturation.^18^ Highly specific Mbs have been engineered by introducing mutations in solvent-accessible residues of the Fn3 scaffold that make up two distinct binding paratopes; the “loop” paratope is along the BC, DE, and FG loops, while the “side” paratope can contribute to target binding through residues on the β-sheet surface (Figure 1B).^17,19^ The tertiary structure of these paratopes affords Mbs the ability to bind a wide range of convex, concave and flat epitopes of target biomarkers, which makes them unique even among other synthetic binding proteins (e.g., affibodies, DARPin and anticalin contain primarily concave paratopes while nanobodies contain primarily convex paratopes).^17^ By modifying these regions through directed evolution, large libraries of diverse Mb variants can be generated and screened for selective binding toward a wide array of protein conformations and functional states.^20^ The modular nature of monobodies, combined with their inherent thermal stability and robustness, makes them particularly advantageous for use in physiologically complex media.^19,21,22^ Furthermore, their capacity to maintain functionality across a broad range of temperatures and in the presence of denaturing agents makes them promising biorecognition elements for diagnostic tools.^23–25^

**Figure 1:**
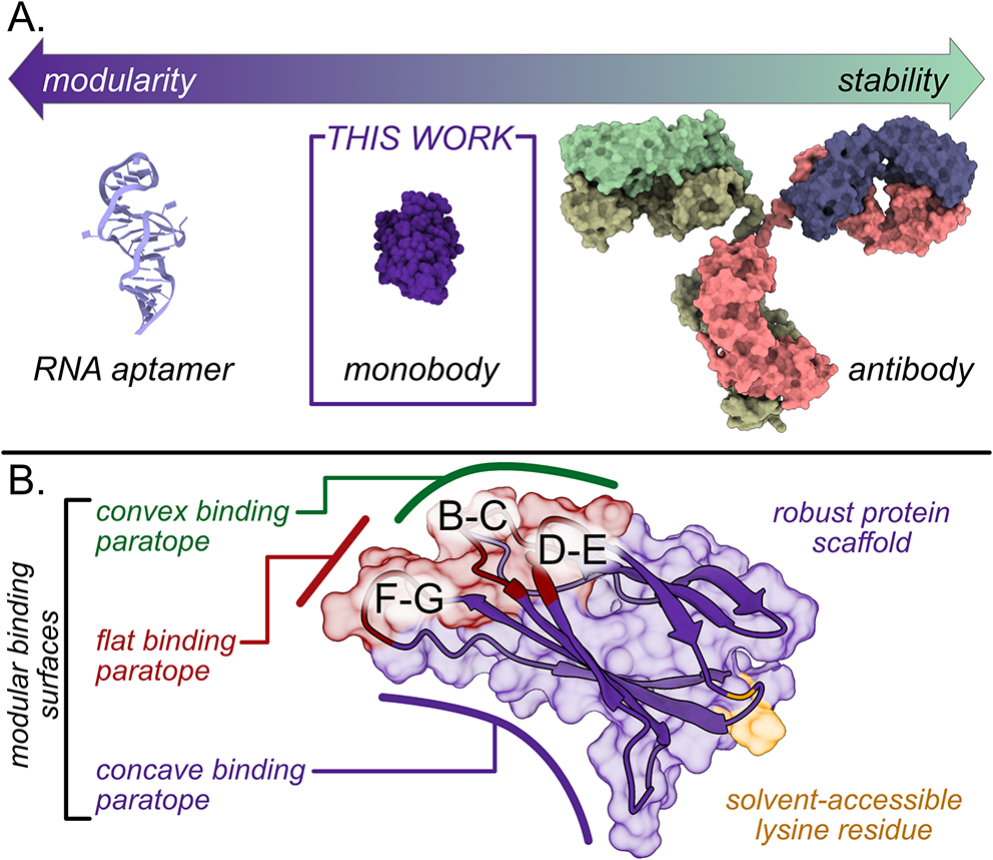
(A) A schematic illustrating the size versus modularity of various biorecognition elements used in electrochemical biosensing. Synthetic proteins, such as monobodies, offer a balance of stability and modularity compared to antibodies and DNA/RNA aptamers. (B) Illustration of the diverse binding paratopes and solvent-accessible lysine residues of the fibronectin type III (Fn3) domain (PDB ID: 1TTG).

The modular binding surface of Mbs makes them especially well-suited for applications, such as reagentless electrochemical biosensors, that require immobilization onto either metallic or biotic surfaces.^26^ For this reason, Mb mutants are often screened for target affinity with an “anchor design” strategy using either phage-, mRNA-, or bacterial surface display.^27^ Using this approach, Mbs have been engineered for preclinical applications to target numerous protein biomarkers, such as Bcr-Abl kinase, VEGFR-2, and hEphA2, which are related to myelogenous leukemia, glioblastoma angiogenesis, and melanoma, respectively.^28–31^ Most biosensors that utilize Mbs as biorecognition elements are fluorescence-based and are capable of achieving dissociation constants in the low-nanomolar range.^25^ Despite their successful use in spectroscopic sensing applications, there are limited examples of Mbs being used in electrochemical biosensors or at an electrode interface.^24^ Based on their potential suitability for surface-bound applications, we hypothesize that Mbs can be directly immobilized onto an electrode surface while maintaining their binding affinity for a target protein analyte, thereby further expanding the potential applications of these versatile recognition elements in biosensing technology.

Herein, we report an electrochemical biosensor using an engineered monobody as the biorecognition element to detect lysozyme as a model protein biomarker. As shown in Figure 2, an engineered monobody is immobilized directly onto the surface of aryl-*N*-hydroxysuccinimide (NHS) ester-functionalized glassy carbon electrodes (GCEs) through reaction with solvent-accessible lysine residues. Incubation of the modified electrodes in the presence of lysozyme results in binding of the target analyte, thereby impeding electron transfer of a solvated redox-active reporter molecule, (ferrocenylmethyl) trimethylammonium chloride (FcNMe_3_). This decreased accessibility to the electrode can be measured by square wave voltammetry (SWV), so that binding of a target protein results in a decrease peak SWV current density for the redox molecule. As a model analyte, lysozyme is a large, soluble protein that has been implicated as a biomarker for a variety of conditions, including breast cancer and Alzheimer’s disease.^32,33^ Specifically, lysozyme levels are increased in Alzheimer’s disease, where the protein interacts with amyloid-β, a peptide that drives plaque formation.^33^ We demonstrate the ability of a Mb-based electrochemical biosensor to detect lysozyme at physiologically relevant concentrations and show that it remains stable through continuous use. Additionally, we show that the Mb-based sensor maintains high sensitivity in a complex biological medium. To our knowledge, this work is the first demonstration of a MB-based biorecognition element in an electrochemical biosensor. This study highlights the potential of monobodies to create highly selective and sensitive biosensors for protein detection.

**Figure 2:**
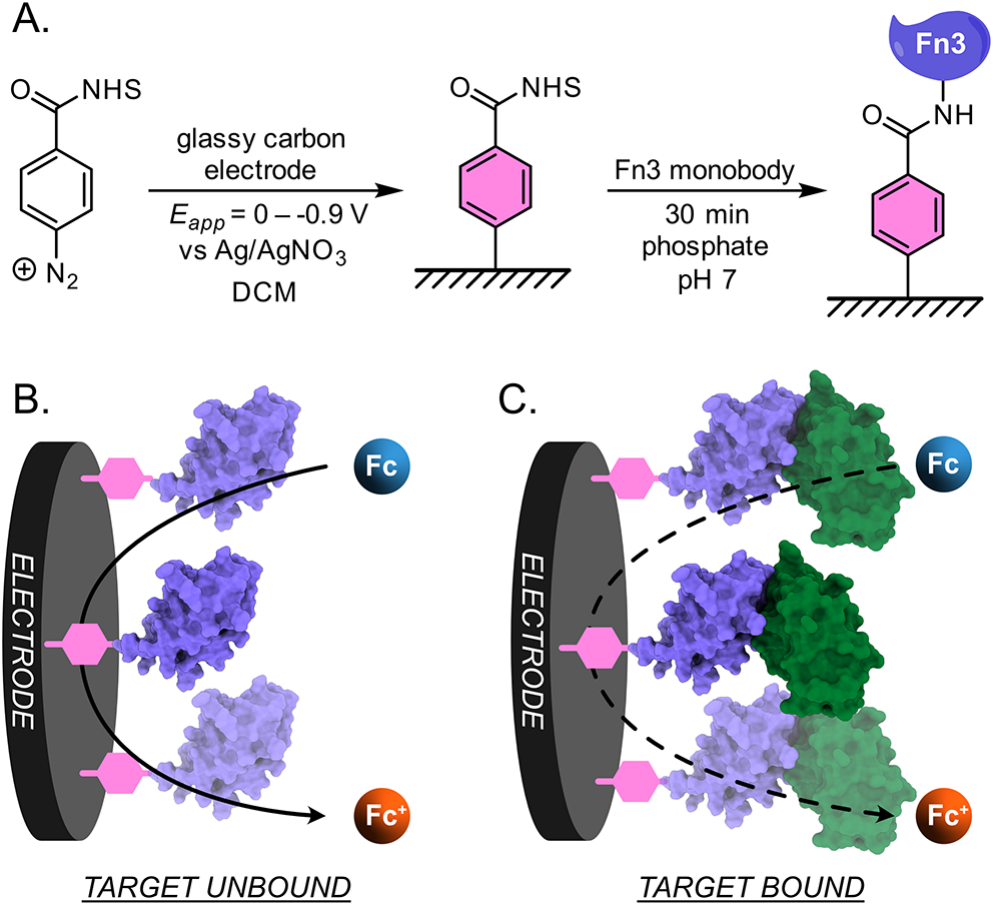
(A) Scheme describing the procedure used to functionalize a glassy carbon electrode with an engineered Fn3 monobody binding protein (purple), including (step 1) swept potential electrochemical grafting from a solution of 4 mM aryldiazonium NHS ester in dichloromethane with 100 mM NBu_4_PF_6_, and (step 2) incubation of the grafted electrode in a solution of 120 μg mL^–1^ Fn3 monobody. (B) Illustration of the Fn3-modified electrode using a dissolved ferrocene species as a redox probe; in the absence of bound target protein, ferrocene has relatively free access to the electrode surface. (C) Upon binding of the target protein (green), the access of ferrocene to the electrode is restricted; this restriction can be quantified using square wave voltammetry.

## Material and Methods

Reagent-grade chemicals were utilized throughout this study. Phosphate buffered saline (PBS), (ferrocenylmethyl)­dimethylamine, *N*-hydroxysuccinimide, 1-ethyl-3-(3-(dimethylamino)­propyl) carbodiimide, and chicken egg white lysozyme were purchased from Sigma-Aldrich. Bovine serum albumin (BSA) was purchased from Abcam. All other chemicals were purchased from Fisher Scientific and used as received without further purification. Electrochemical measurements were performed using a Biologic potentiostat (VSP, 5 channel) with a SCE reference electrode, a Pt wire counter electrode (Bioanalytical Systems, West Lafayette, IN), and a 3 mm glassy carbon working electrode (CH instruments).

### Protein Production

The human fibronectin type-II domain III plasmid FL063 sequence with a cysteine at position 102 was designed using IDTDNA. The pCT and Pet22b plasmids were obtained from IDTDNA and expressed in T7 (*E*. *coli*) cells (NEB Cat: C2566H). LB and kanamycin solution were used to cultivate starting cultures of T7 cells, which were grown overnight. These cultures were then transferred to 2 L cell cultures without antibiotics and incubated overnight at room temperature on an orbital shaker at 250 rpm. The induction with IPTG for 16 h was revealed to yield the highest protein concentration by SDS-PAGE results. Cell lysis was performed using the French press mechanism, and protein purification was executed using HisPur Cobalt columns in an FPLC. Amicon filters of 10 kDa were utilized for further concentration of the purified proteins. The expression of the desired protein and the yield of production were verified by SDS-PAGE. A Nanodrop was employed for the determination of the protein concentration.

### Engineering Strategy and Library Construction

The initial diversity of the hydrophilic human fibronectin monobody variant library was approximately 10^9^ in *S*. *cerevisiae* yeast. This yeast surface display library was previously screened for high-affinity lysozyme binders using magnetic and fluorescence-activated cell sorting.^18^ In brief, 20 × 10^9^ yeast cells were pooled—resulting in a 20-fold abundance of each of the 10^9^ unique monobody variants on average—and subjected to three rounds of depletion sorting to remove nonspecific “sticky” variants and any that bound to magnetic bead reagents. Following depletion, the library was enriched through three rounds of positive selection magnetic bead sorting (using biotinylated lysozyme immobilized on streptavidin-coated magnetic beads) and three rounds of fluorescent-activated cell sorting (FACS) using progressively lower concentrations of lysozyme (50 nM, then 5 nM, then 500 pM). Sequencing of the highly enriched population revealed 25 unique monobody sequences. For our biosensor application, where the monobody is immobilized on an electrode surface, the ideal clone was selected based on its ability to bind.

### Binding Affinity Determination and Validation

Affinity titration was performed to determine the binding affinity of the lysozyme-binding monobody variant FL063. For this assay, FL063 was induced for expression on the surface of *EBY100* yeast. A C-terminal cMyc tag was used to quantify monobody expression levels, while fluorescently labeled lysozyme was incubated with the monobody-displaying yeast at lysozyme concentrations of 0, 0.2, 0.4, 0.5, 2, 20, and 100 nM. The observed median fluorescence intensity (*F*) for each concentration was fit to the equation

$$F=F_{\min}+\frac{\left(F_{\max}-F_{\min}\right)\left[\mathrm{Lyso}\right]}{\left(\left[\mathrm{Lyso}\right]+K_{d}\right)}$$

where [Lyso] is the lysozyme concentration, *K*_d_ is the binding constant, *F*_max_ is the maximum fluorescent signal (from the 50 nM sample), and *F*_min_ is the baseline signal (from the 0 nM sample). In addition, the FL063 variant was tested for binding against three nonlysozyme targets (human transferrin, rabbit IgG, and goat IgG) at 500 nM. No fluorescent signal above background was observed for these targets, confirming the high specificity of the FL063 monobody.

### Synthesis of 4-Carboxybenzenediazonium BF_4_

4-Carboxybenzenediazonium tetrafluoroborate was prepared as described previously.^34^ 4-aminobenzoic acid (2.74 g, 20.0 mmol) was dissolved in a solution of fluoroboric acid (48%, 14.6 g, 80 mmol) and water (20 mL) at 40 °C to ensure dissolution. A solution of sodium nitrite (1.46 g, 21.2 mmol) in water (4 mL) was added dropwise over several minutes to the stirred aminobenzoic acid solution at 0 °C. The diazonium product precipitated upon addition of sodium nitrite, and the resulting white solid was collected by vacuum filtration with and washed with cold diethyl ether (10 mL, five times). Excess solvent was removed from the solid product under reduced pressure to yield 1.24 g of the desired diazonium salt (26% yield). ^1^H NMR (400 MHz, DMSO) δ (ppm); 8.42 (2H, d), 8.78 (2H, d), 14 (H, s).

### Synthesis of Aryldiazonium NHS Ester

4-[[(2,5-Dioxo-1-pyrrolidinyl)­oxy]­carbonyl]­benzene-diazonium was prepared similar to the reaction conditions described previously.^35^ Aryl diazonium salt (0.100 g), 1-ethyl-3-(3-(dimethylamino)­propyl)­carbodiimide (EDC) (0.115 g) and *N*-hydroxysuccinimide (NHS) (0.215 g) were combined in anhydrous CH_2_Cl_2_ (12 mL) at 0 °C. The resulting solution was stirred for 16 h at room temperature. The organic solution was successively washed with 1 M HCl (aq) then a saturated aqueous solution of NaHCO_3_. The organic fraction was dried over MgSO_4_ and the solvent was removed under reduced pressure resulting in a reddish-orange viscous liquid. ^1^H NMR (400 MHz, DMSO-*d*_6_): δ 2.89 (s, 4H), 8.20–7.47 (m, 4H).

### Synthesis of Water-Soluble (Ferrocenylmethyl)­trimethylammonium Chloride (FcNMe_3_)

(Ferrocenylmethyl)­trimethylammonium chloride (FcNMe_3_) was prepared as previously described.^36^ Chloromethane (9.0 mL of 1 M solution in *tert*-butyl ether, 0.5 mmol) was added so a stirring solution of (ferrocenylmethyl)­dimethylamine (0.10 g, 0.41 mmol) in acetonitrile (10 mL). The reaction mixture was stirred at room temperature for 24 h. The resulting red-orange precipitate was collected by vacuum filtration. The remaining supernatant was added slowly to a stirring solution of diethyl ether (100 mL), and the resulting precipitate was collected by vacuum filtration. The combined solid product was washed with diethyl ether (100 mL) and excess solvent was removed under reduced pressure, resulting in an orange solid product (0.089 g, 75% yield). The hygroscopic product was stored in a dry desiccator. ^1^H NMR (300 MHz, D_2_O): δ 2.91 (s, 9H), 4.24­(s, 5H), 4.35 (s, 2H), 4.39 (s, 2H), 4.47 (d, 2H).

### Electrochemical Grafting of Aryl NHS Ester

Glassy carbon electrodes were functionalized with aryl NHS ester groups through electrochemical grafting of the corresponding aryl diazonium salts. Prior to grafting, GCEs were polished with a cloth polishing pad and 1.0, 0.3, and 0.05 μm alumina slurries sequentially to ensure a clean surface. Electrochemical grafting was done by performing cyclic voltammetry using a 4 mM solution of 4-[[(2,5-dioxo-1-pyrrolidinyl)­oxy]­carbonyl]­benzene-diazonium in CH_2_Cl_2_ with 100 mM TBAPF_6_. For electrochemical grafting, the potential was swept 10 times (five cycles) between 0 V and −0.9 V vs Ag/AgNO_3_ at 100 mV s^–1^. After electrochemical grafting, modified GCE electrodes were rinsed with DI water and immediately modified with binding protein.

### Immobilization of FL063 Binding Protein onto GCE

Immediately after electrochemical grafting of GCEs with aryldiazonium, the NHS-modified GCEs were incubated in a stirred solution containing 10 μM (0.12 mg mL^–1^) FL063 binding protein in phosphate buffered saline at pH 7 and 25 °C for 30 min. Electrodes were rinsed with DI water prior to use for sensing experiments.

### Sample Analysis and Electroanalytical Methods

Square wave voltammetry (SWV) was performed for analytical measurements on FL063-modified GC electrodes (either with or without lysozyme incubation step) with 20 mV pulse height, 5 mV step height, and 10 ms pulse width using a solution of FcNMe_3_ (1 mM) in 100 mM phosphate buffer at pH 7 and 25 °C. For sample analysis, FL063-modified electrodes were incubated for 30 min in 100 mM phosphate buffer solutions containing variable concentrations of lysozyme (or bovine serum albumin for control experiments) at pH 7 and 25 °C. After incubation, electrodes were rinsed with DI water and tested analyzed by SWV. For experiments in canine serum, FL063-modified electrodes were incubated for 30 min in 100 mM phosphate buffer solutions with 30 vol % canine serum and variable concentrations of lysozyme (or BSA for control experiments).

## Results and Discussion

Monobodies can be engineered through construction of combinatorial phage-display libraries designed to diversify their binding regions. To ensure a robust library of binding proteins without altering the core scaffold, engineering efforts are commonly focused on diversifying (1) the B–C, D-E, and F-G loops and (2) two loops at opposing ends of the Fn3 scaffold along with the intermediate β-sheet surface. These approaches create a broad range of target-binding topographies, enabling monobodies to bind diverse target epitopes effectively. We selected three engineered fibronectin monobody sequences from an existing lysozyme binder library and amplified their DNA sequences using PCR. Plasmids were constructed primarily through two methods: Gibson Assembly for yeast surface display pCT vectors, and digestion and ligation for protein expression pET vectors. The corresponding constructs were verified via Sanger sequencing and expressed in yeast cells for surface display binding studies or in bacterial cells for protein production.

Diversity of the initial hydrophilic human fibronectin monobody variant library was approximately 10^9^ in *S*. *cerevisiae* yeast. This yeast surface display library was previously screened for high-affinity lysozyme binders using magnetic and fluorescence-activated cell sorting.^18^ In brief, 20 × 10^9^ yeast cells were pooled—resulting in a 20-fold abundance of each of the 10^9^ unique monobody variants on average—and subjected to three rounds of depletion sorting to remove nonspecific “sticky” variants and any that bound to magnetic bead reagents. Following depletion, the library was enriched through three rounds of positive selection magnetic bead sorting (using biotinylated lysozyme immobilized on streptavidin-coated magnetic beads) and three rounds of fluorescent-activated cell sorting (FACS) using progressively lower concentrations of lysozyme (50 nM, then 5 nM, then 500 pM). Sequencing of the highly enriched population revealed 25 unique monobody sequences. Among these monobody sequences, preliminary binding assays suggested that three variants (Fn3G, Fn3103, and FL063) exhibited selective binding for lysozyme.

To determine the precise binding affinity of the three down-selected variants to lysozyme, we performed flow cytometry-assisted affinity titration using the Fn3G, Fn3103, and FL063 variants. For this assay, binding proteins were induced for expression on the surface of *EBY100* yeast. A C-terminal cMyc tag was used to quantify monobody expression levels, while fluorescently labeled lysozyme was incubated with the monobody-displaying yeast at varying lysozyme concentrations. The observed median fluorescence intensity (*F*) for each concentration was fit to the equation

1 $$F=F_{\min}+\frac{\left(F_{\max}-F_{\min}\right)C_{\mathrm{Lys}}}{C_{\mathrm{Lys}}+K_{d}}$$

where *C*_Lys_ is the lysozyme concentration, *K*_d_ is the binding constant, *F*_max_ is the maximum fluorescent intensity (from a 50 nM lysozyme sample), and *F*_min_ is the baseline intensity. The resulting mean fluorescence values (shown in Figure S4) are in good agreement with eq 1 and reveal *K*_d_ values of 298.7, 232.5, and 1.7 nM for the Fn3G, Fn3103, and FL063 variants, respectively. Based on these results, FL063 was selected as the lysozyme biorecognition element for all subsequent experiments. In addition, FL063 was tested for binding against three nonlysozyme targets (human transferrin, rabbit IgG, and goat IgG) at 500 nM to serve as negative control experiments. No fluorescent signal above background was observed for these targets, confirming the high specificity of the FL063 monobody.

With the engineered biorecognition element in hand, we next sought to immobilize FL063 onto an electrode surface while ensuring that its binding domain remained intact and accessible to dissolved lysozyme. This was accomplished by a two-step process of (1) functionalizing glassy carbon electrodes with *N*-hydroxysuccinimide (NHS) ester groups, and (2) reacting the resulting surface-bound NHS esters with a solvent-accessible lysine residue of FL063 (as illustrated in Figure 2). Electrodes were functionalized with NHS esters by a well-established method of electrochemically grafting 4-substituted aryl diazonium.^37–40^ Cyclic voltammetry was performed using a solution of 4-[[(2,5-dioxo-1-pyrrolidinyl)­oxy]­carbonyl]­benzene-diazonium (4-phenyldiazonium-NHS ester) at reducing potentials (−0.9 V vs Ag/AgNO_3_) to generate the corresponding aryl radical species that spontaneously form covalent bonds with the GCE surface. Additional details on characterization of grafted electrodes are provided in the Supporting Information. The resulting NHS-functionalized electrodes were incubated in a solution containing FL063 to allow for reactivity between the modified electrode and solvent-accessible lysine residues of the binding protein. The resulting electrode surface coverage was estimated to be ∼ 23% by comparison of experimental and simulated SWVs (Figure S4).

It should be noted that FL063 contains three solvent-accessible lysine residues, which could lead to three possible orientations once anchored to the electrode surface. The extent to which surface conjugation influences the ability of FL063 to bind lysozyme depends on the location of the anchoring lysine residue relative to the functional paratope. Previous studies have demonstrated that surface conjugation of larger affinity proteins via residues distal to the paratope generally preserve their binding function.^41–43^ In contrast, surface conjugation through residues located within or near the paratope often obstruct access to the paratope while impeding or entirely preventing target binding. Structural analysis of FL063 reveals one lysine residue is located within the target paratope (near the B–C loop), such that conjugation is expected to diminish binding affinity; however, the remaining two lysine residues are located at distal positions along the A strand and the E-F loop where conjugation is not expected to alter target binding. Assuming that all residues have similar accessibility, we would expect the majority of immobilized protein to be oriented so that the desired paratope is accessible to bind dissolved lysozyme.

To ensure electrode surface modification with FL063, we performed square wave voltammetry (SWV) on aqueous solutions of (ferroceneylmethyl)­trimethylammonium chloride (FcNMe_3_) as a water-soluble redox probe using either bare electrodes or aryl-NHS ester-grafted electrodes before and after incubation with FL063 (Figure S2). In a solution of FcNMe_3_, electrodes modified with only the NHS ester did not show a significant difference in peak SWV current density (*j*_p_) when compared to a bare GCE (683 ± 6 μA cm^–2^ for bare electrodes compared to 680 ± 20 μA cm^–2^ for NHS-ester grafted electrodes). After incubation with a solution containing FL063 (followed by rinsing to remove any physically adsorbed protein), NHS-grafted electrodes exhibited smaller *j*_p_ values (644 ± 4 μA cm^–2^) consistent with decreased accessible electrode surface area.^44^ After incubating FL063-modified electrodes in a solution containing 45 μM lysozyme, the peak current density decreased further to 370 ± 20 μA cm^–2^ (Figure 3A). These results indicate that the FL063 binding protein is bound to the electrode surface and that bound protein is capable of binding to lysozyme. To ensure binding specificity for lysozyme, FL063-modified electrodes were incubated with a 45 μM solution of bovine serum albumin (BSA) as a negative control. Subsequent SWVs revealed no change in peak current densities for the BSA-incubated electrodes, as shown in Figure 3B.

**Figure 3:**
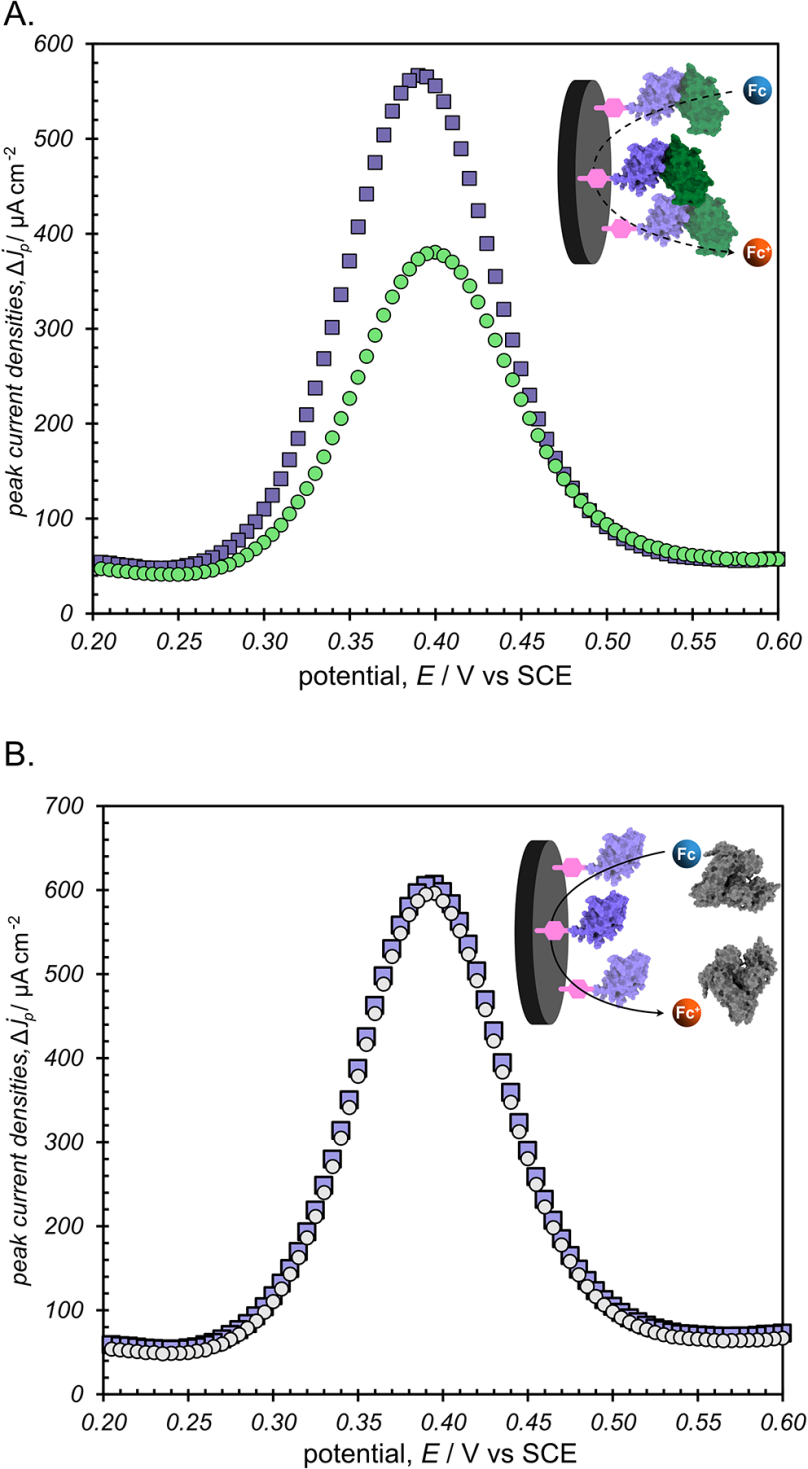
Representative square wave voltammograms of 1 mM FcNMe_3_ using FL063-modified carbon electrodes either (A) before (purple squares) and after (green circles) incubation in a solution containing 40 μM lysozyme, or (B) before (purple squares) and after (gray circles) incubation in a solution containing 40 μM bovine serum albumin (BSA) as a negative control. Experiments were performed with a pulse time of 10 ms, a step height of 5 mV and an amplitude of 20 mV, using 100 mM phosphate buffer at pH 7 and 25 °C.

It should be noted that FcNMe_3_ was selected as the redox reporter in this study due to its well-established high solvated storage stability, high aqueous solubility for both its oxidized and reduced forms, and high electrochemical reversibility. In addition to the highly soluble ferrocene, anthraquinone-2-sulfonate (AQS) was investigated as an organic redox probe with a substantially lower reduction potential and opposite electrostatic charge as the FcNMe_3_ cation. The binding behavior of FL063-modified electrodes after incubation with lysozyme (and corresponding decrease in peak SWV current) when using AQS as a redox probe is similar to that observed with FcNMe_3_ (Figure S7). These results suggest that monobody-based biosensors are at least partially redox probe-agnostic so that the solvated redox species can be selected to minimize the impact of undesired electrochemical interferants (such as ascorbate and acetaminophen). Following the demonstration of specific binding to lysozyme, we next sought to determine the biosensor sensitivity and detection limits across a broader range of lysozyme concentrations.

To determine the resolution of the biosensor, FL063-modified electrodes were incubated in solutions with variable concentration of lysozyme. A plot of sample lysozyme concentration versus the corresponding change in peak SWV current density (Figure 4) shows that peak SWV current density decreases with increasing lysozyme concentration until reaching saturation beyond 20 μM. This behavior is consistent with a Langmuir adsorption isotherm and reveals a linear range up to 1 μM. It should be noted that the difference in peak SWV current density (Δ*j*_*p*_) here is defined as the peak SWV density (*j*_*p*_) observed using a solution of 1 mM FcNMe_3_ prior to incubation with lysozyme minus *j*_*p*_ observed after incubation with lysozyme. In contrast to electrodes incubated with lysozyme, control experiments using BSA revealed no significant change in the peak current density difference between the binder-modified electrode and those exposed to the albumin solution, further demonstrating the specificity of this monobody-based sensor for lysozyme detection.

**Figure 4:**
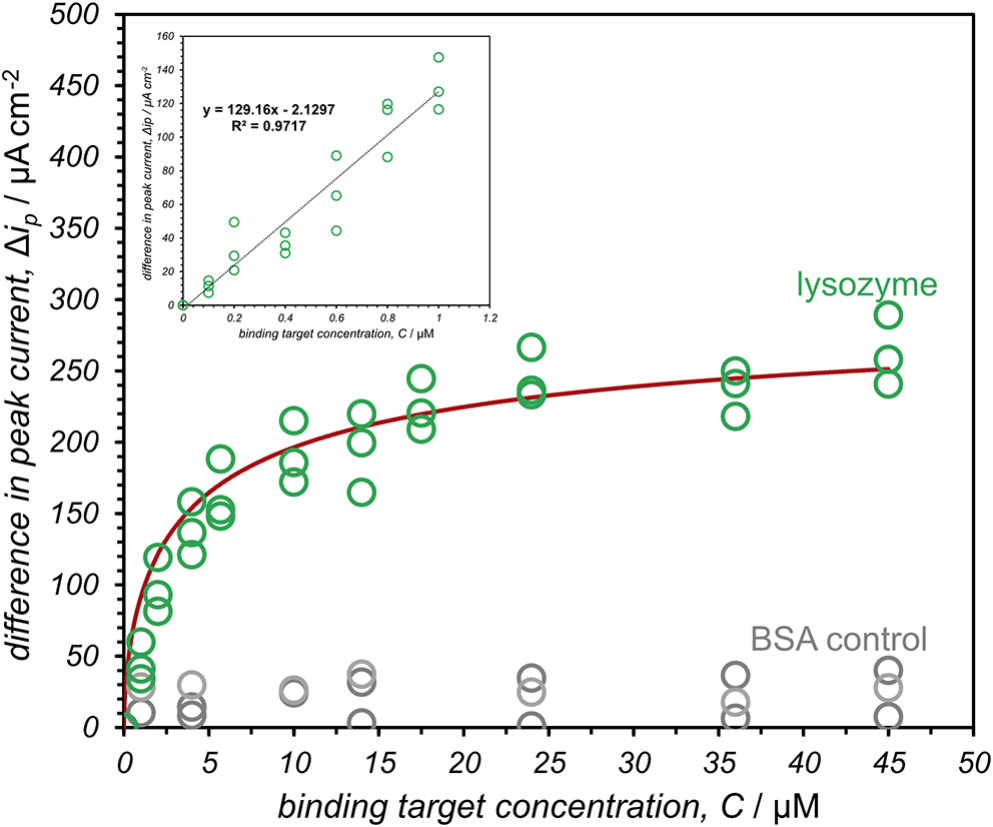
Langmuir isotherm model for FL063-modified electrodes after incubation in various concentrations of lysozyme (green) and BSA (gray), where lysozyme saturation was reached at 42 μM. Differences in peak SWV current density were measured using a solution of 1 mM FcNMe_3_ in 100 mM phosphate buffer. The inset plot shows the linear response region of the curve between 0.2 μM and 1 μM lysozyme. Experiments were performed with a pulse time of 10 ms, a step height of 5 mV and an amplitude of 20 mV, using 100 mM phosphate buffer at pH 7 and 25 °C. Each lysozyme sample (and BSA control sample) concentrations were measured in triplicate with six total electrodes (three for lysozyme samples and three for BSA control samples).

The limit of detection (LOD) for the monobody-modified electrochemical biosensor was determined to be 0.09 ± 0.01 μM lysozyme (shown in Figure 4 inset), with the limit of quantification (LOQ) established at 0.29 ± 0.03 μM. These parameters were derived utilizing the standard deviation method, where the LOD was calculated by tripling the standard deviation of the blank readings divided by the slope of the linear response range. Similarly, the LOQ was defined as ten times one standard deviation of the blank divided by the same slope.^45^ The calibration curve, (Figure 4 inset) demonstrates a robust linear relationship between the difference in peak current and the concentrations of lysozyme across a range spanning from 0.1 μM to 1.0 μM. This helps in understanding the binding equilibrium of the biosensor as it can be directly correlated with the sensitivity of the interface.

Assuming that the distribution of immobilized FL063 is approximately uniform on the electrode surface, the binding data was fit to a Langmuir adsorption isotherm (Figure 4).^46,47^ In the context of the electrochemical data generated, the Langmuir adsorption isotherm equation can be rewritten as

$${\Delta i}_{p}=\frac{\left({\Delta i}_{p,\max}KC^{X}\right)}{1+KC^{X}}$$

Where Δ*j*_p_ (μA cm^–2^) is the observed peak SWV current density difference, Δ*j*_p,max_ (μA cm^–2^) is the maximum difference in peak SWV current density due to a one-on-one binding with the monobody on the electrode surface, *C* (nM) is the lysozyme concentration in solution, *K* (μM^–1^) is the Langmuir adsorption coefficient, and *X* is a concentration-dependent factor to correct for surface heterogeneity. The fitted value of Δ*j*_p_ = 295 μA cm^–2^ using a modified Langmuir model is consistent with the observed maximum difference in peak current and suggests a slightly higher maximum lysozyme binding capacity than measured experimentally. Additionally, the Langmuir model is consistent with an adsorption coefficient of *K* = 0.25 μM^–1^ and a homogeneity factor *X* = 0.95. A value approaching one for the exponential term, *X*, suggests a homogeneous surface coverage of bound FL063 on the electrode surface and indicates that binding of lysozyme to one FL063 protein on the surface does not impact binding of adjacent sites. The measured adsorption coefficient indicates a significantly lower sensitivity of electrode-bound FL063 compared to the yeast displayed binding protein; this may be due to the location of anchoring lysine residues relative to the binding paratope. Despite reduced sensitivity, the apparent lack of binding to BSA suggests that electrode-bound FL063 maintains a high selectivity for lysozyme.

To assess the compatibility of the sensor in complex physiological fluids of larger mammals,^45,48^ the binding equilibrium and sensitivity of FL063-modified biosensors toward lysozyme were evaluated in dilute 30 vol % canine serum. Serum samples were doped with known concentrations of lysozyme, and the electrodes functionalized with monobodies were incubated in the corresponding solutions for 15 min before each electrochemical analysis. The resulting changes in peak SWV current density as a function of lysozyme concentration (Figure 5) indicates a Langmuir-type binding response, mirroring experimental trends of lysozyme in phosphate buffer. Unlike experiments in phosphate buffer, a significant amount of nonspecific adsorption was observed that appeared to be caused by the aggregation of the serum matrix with the addition of an exogenous protein. The result was a decrease in peak current density upon addition of either lysozyme or BSA (as a negative control) up to 50 μg mL^–1^ of added protein mass concentration. Incubation in protein concentration higher than 50 μg mL^–1^ resulted in typical binding isotherm behavior for lysozyme, while incubation in increasing concentrations of BSA did not significantly change peak SWV current density. We interpret this increased Δ*j*_*p*_ at low protein concentration as nonspecific adsorption of serum contents onto unfunctionalized regions of the electrode surface. Furthermore, the nonspecific adsorption observed upon the addition of BSA to the diluted canine serum may be due to other nontarget interactions with proteins inherently present in the serum, as the clinical history of donor canines was not established. This interaction could account for the observed alterations in peak currents for the BSA control assays at low protein concentrations.

**Figure 5:**
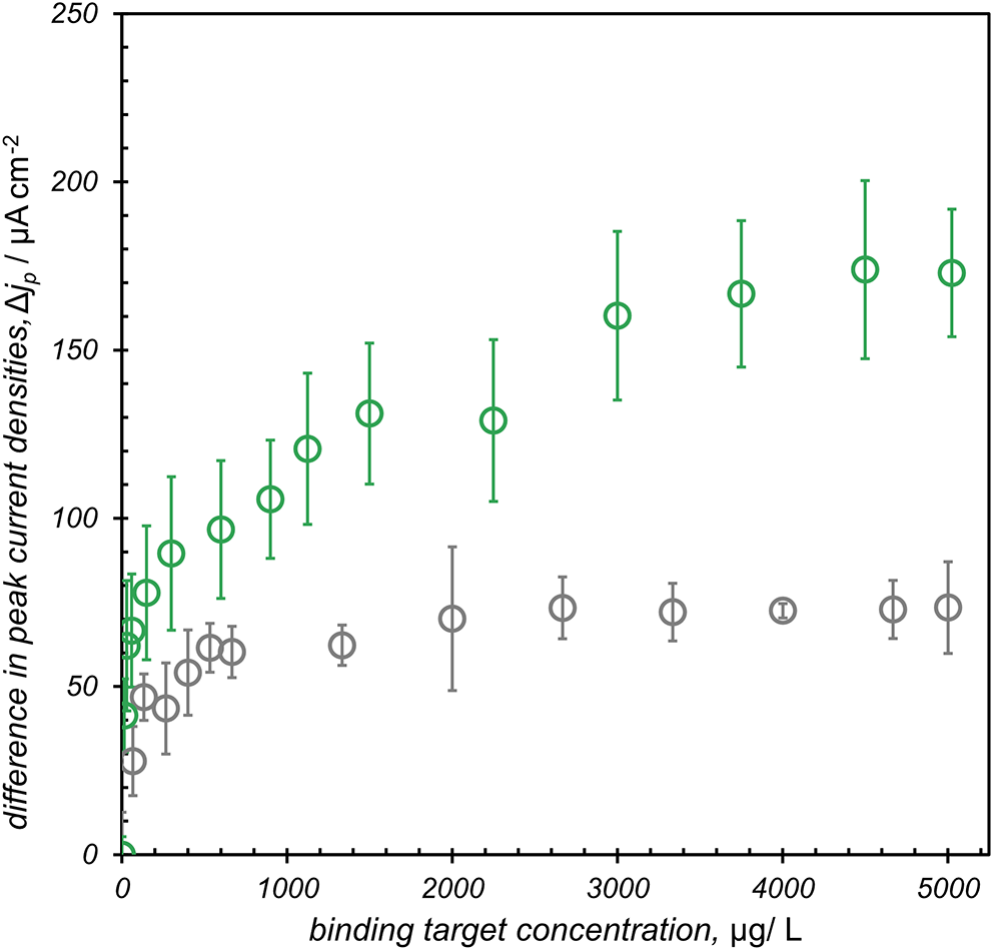
Response of FL063-modifed electrodes to incubation in 30 vol % canine serum solutions containing variable concentrations of lysozyme (green) or BSA (gray). SWV measurements were made using a solution of 1 mM FcNMe_3_ after incubation in the serum sample for 10 min. Experiments were performed with a pulse time of 10 ms, a step height of 5 mV and an amplitude of 20 mV, using 100 mM phosphate buffer at pH 7 and 25 °C. Error bars represent one standard deviation from mean values (*n* = 3).

Evaluating the reproducibility and stability of electrochemical biosensors is essential, as evidenced by studies showing that different immobilization strategies can result in varying degrees of signal retention over multiple testing cycles.^26^ Consequently, we next aimed to determine the consistency of consecutive measurements as a preliminary metric for device stability. FL063-modified electrodes were incubated with lysozyme and subjected to 100 continuous SWV scans at room temperature. Plots of peak SWV current density versus scan number (Figure 6) indicates no statistically significant change in the signal over 100 scans. It should be noted that a slight degradation in the peak SWV current density is observed in both the blank control and the lysozyme-bound electrodes, but the signal loss is consistent for both sets of electrodes. This suggests that there may be some degradation of immobilized FL063 followed by subsequent adsorption onto the electrode surface. Nevertheless, the pervasive signal degradation is relatively small (2.1% decrease in peak SWV current density) and occurs entirely in the first 10 scans. After this initial break-in phase, the sensor demonstrated <3% signal loss over the remaining 90 measurements. This highlights the durability and consistent performance of this FL063 monobody-based electrochemical biosensor over numerous scan cycles.

**Figure 6:**
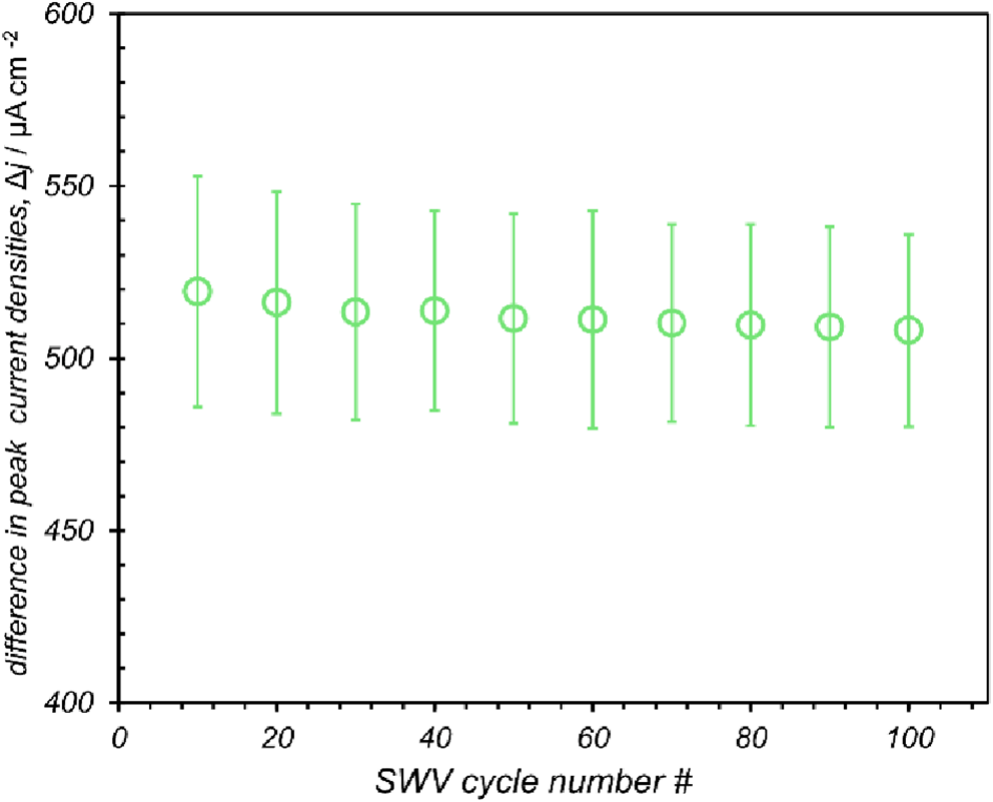
Plot of peak current density signal during continuous SWV scanning (over 90 min) for FL063-modified electrodes in a 1 mM FcNMe_3_ solution after incubation in 10 μM lysozyme. Experiments were performed with a pulse time of 10 ms, a step height of 5 mV and an amplitude of 20 mV, using 100 mM phosphate buffer at pH 7 and 25 °C. Error bars represent one standard deviation from mean values (*n* = 3).

While the present study focused on the electrical stability of the FL063 monobody-modified electrodes, as demonstrated by consistent signals over 100 measurement cycles, systematic assessment of long-term storage stability and interbatch reproducibility remains an important goal for future work. The intrinsic thermal stability of monobody scaffolds, which enables retention of structure and function under diverse environmental conditions, provides a promising foundation for biosensor durability.^18,49,50^ However, extended shelf life studies and batch-to-batch variability evaluations will be necessary to ensure reliable sensor performance in real-world applications.

## Conclusion

We have demonstrated a novel strategy for detecting the model biomarker, lysozyme, using an engineered monobody-modified biosensor. The engineered monobody, FL063, was covalently attached to the electrode surface through reaction with NHS ester functional groups adhered via electrochemical grafting. This approach is a straightforward and effective strategy for creating a versatile biosensor that can be engineered for various target biomolecules. The resulting lysozyme biosensor exhibited a LOD of 0.09 μM and a LOQ of 0.29 μM with a linear response range up to 1 μM lysozyme. The biosensor maintained high specificity with minimal nonspecific adsorption in canine serum as a model complex matrix. Ongoing studies are aimed at mitigating any nonspecific adsorption of coexistent proteins from complex media to maximize sensitivity of the biosensor in practical applications. Additionally, the biosensor demonstrated robust electrical stability in preliminary continuous use studies by maintaining consistent signals over 100 consecutive measurements in a span of 90 min.

The use of monobodies as biorecognition elements offers a unique advantage in terms of modularity and scalable production. While conventional recognition elements, such as antibodies, require labor-intensive development and often suffer from batch-to-batch variability, MBs can be rapidly engineered for novel targets and are reproducibly expressed in *E*. *coli*.^51,52^ This minimizes variability and facilitates the development of multiplexed sensors, where poor performance of a single element can compromise the overall device function. Our results demonstrate that monobody-based electrochemical biosensors are not only robust and selective but also hold considerable potential for the accurate and reliable detection of large biomolecules, while warranting further exploration and validation for multiplexed diagnostic applications.

## Supplementary Material



## Notes

Additional experimental details, electrochemical simulations, representative square wave voltammograms, yeast surface display binding affinity curves, SWVs using anthraquinone-2-sulfonate as an alternative redox probe, comparison of previously reported lysozyme sensors, protein expression details, and flow cytometry data (PDF)

## †

Department of Chemical Engineering, Massachusetts Institute of Technology, Cambridge, Massachusetts 02139, United States
