# Supplementary material for: Utilizing Engineered Monobodies for the Electrochemical Quantification of Lysozyme
Source: Anal Chem. 2025 Dec 14;97(50):27589–97. doi: 10.1021/acs.analchem.5c03304 (PMC12750402; doi:10.1021/acs.analchem.5c03304)
Supplement: Supplementary file 1 [file ac5c03304_si_001.pdf]

Utilizing Engineered Monobodies for the Electrochemical Quantification of Lysozyme

SUPPORTING INFORMATION

Sunanda Dey†, Andrew Naser, Daniel R. Woldring*, David P. Hickey*

Department of Chemical Engineering and Materials Science, Michigan State University, East Lansing, MI, USA

Electrochemical Grafting Conditions

Electrochemical grafting onto glassy carbon electrodes was performed using cyclic voltammetry, applying a potential sweep from 0 V to -0.9 V using a Pt-wire counter electrode and an $Ag/AgNO_3$ reference electrode using a 4 mM solution of 4-phenyldiazonium-NHS ester in dichloromethane. Five CV cycles were applied during electrochemical grafting to ensure sufficient surface coverage for monobody immobilization. Previous studies have shown that the use of excessive cycling (i.e., beyond ~10 cycles) does not improve protein loading as the GCE reaches surface saturation.[1,2]

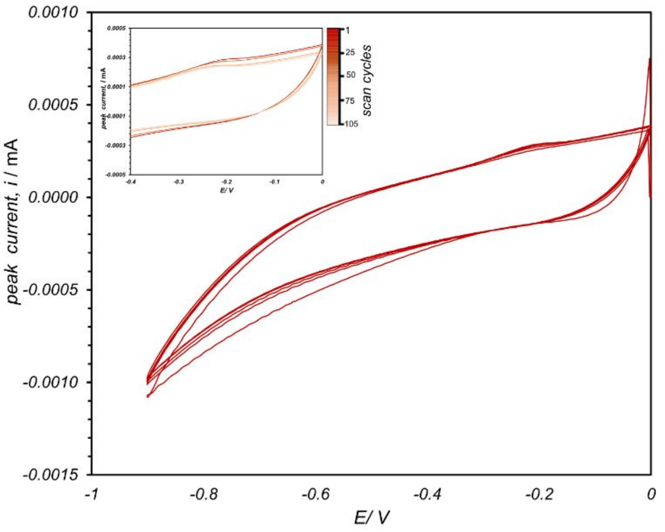

Figure S1. Representative cyclic voltammogram showing electrochemical polymerization of 4-phenyldiazonium-NHS ester on a 3 mm glassy carbon electrode. A total of five scans were performed using a 3 mm glassy carbon working electrode in a 4 mM diazonium NHS ester solution prepared in anhydrous dichloromethane. Grafting was carried out at a scan rate of 100 mV s^{-1} with a $Ag/AgNO_3$ reference electrode at 25 ˚C.

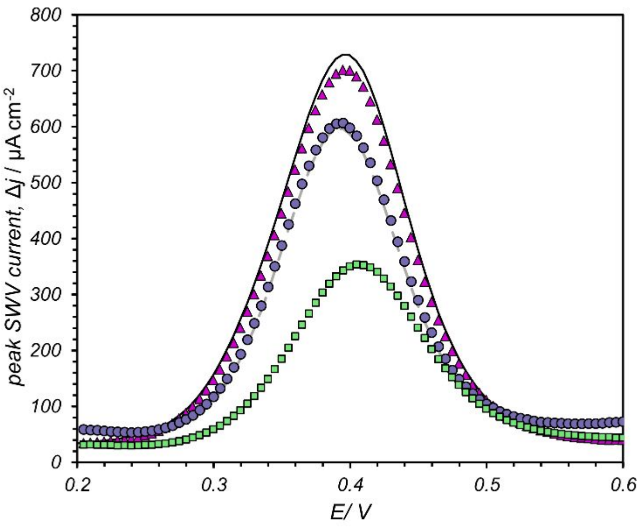

Figure S2. Representative square wave voltammograms of 1 mM ferrocene using bare electrodes (black solid line), and electrodes with NHS ester-grafted (pink triangles), FL063-grafted (purple circles), and FL063-grafted after incubation in 45 μM lysozyme (green). All experiments were performed on FL063-modified 3mm glassy carbon working electrodes (GCE) and a SCE reference electrode, with a pulse time of 10 ms, a step height of 5 mV and an amplitude of 20 mV using 100 mM phosphate buffer at pH 7 and 25 °C.

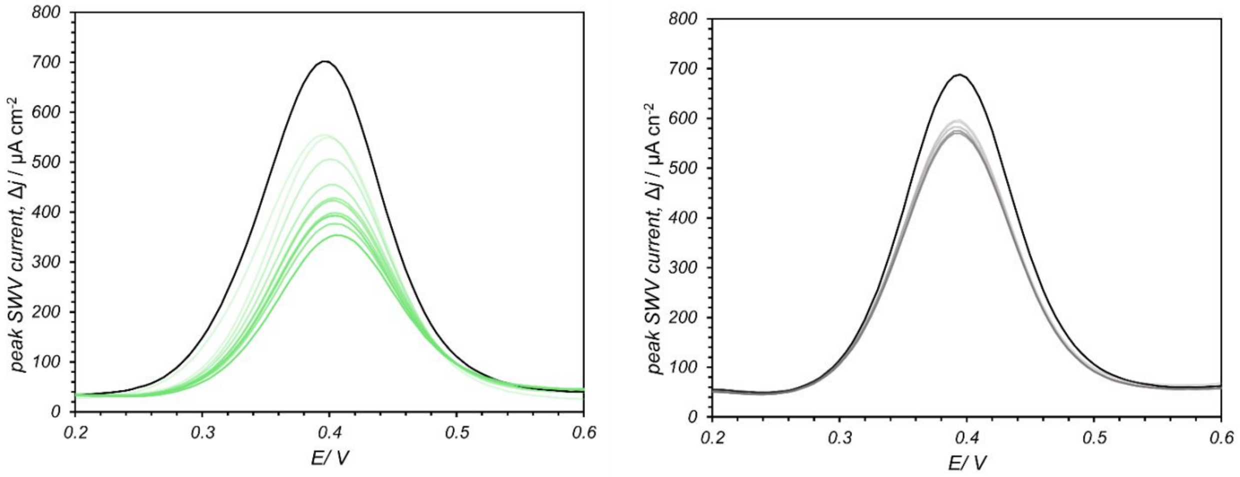

Figure S3. Representative square wave voltammograms compare the electrochemical response of FL063-modified glassy carbon electrodes in 1 mM $FcNMe_3$ after incubation in a solution of lysozyme and BSA control. The left plot illustrates a progressive decrease in peak current with increasing lysozyme concentrations (light to dark green, ranging from 1 µM to 45 µM), while the black voltammogram represents the unmodified electrode control. The plot on the right shows the response of FL063-modified electrodes in the presence of BSA in the supporting electrolyte, where no significant change in peak current is observed with increasing BSA concentrations (light to dark grey), confirming high specificity for lysozyme even in a complex biological matrix. The black plot in both cases represents unmodified electrode control. Experiments were performed with a pulse time of 10 ms, a step height of 5 mV and an amplitude of 20 mV, using 100 mM phosphate buffer at pH 7 and 25 °C.

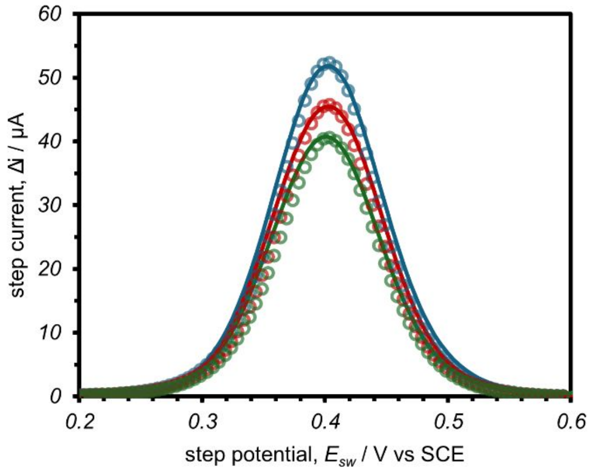

Figure S4. Representative experimental (solid lines) and simulated (open circles) SWVs of 1 mM $FcNMe_3$ with either bare (blue), aryl NHS-grafted (red) or FL063-functionalized (green) glassy carbon electrodes. SWV simulations were performed with either 100% (blue), 87% (red), or 77% accessible electrode surface area, which suggests that electrode functionalization results in ~23% surface coverage of monobody binding protein. Electrochemical simulations were performed using DigiElch 8 using a diffusivity of 8.5×10^{-6} cm^2 s^{-1}, as measured from cyclic voltammetry analysis (data not shown). The projected area of the FL063 (e.g., the footprint of each bound protein on the electrode) is 3694 Å per molecule, which indicates an immobilized monobody surface density of ~6 x 10^{11} molecules cm^{-2}. This surface density is consistent with previously reported surface densities for both immobilized aptamers (typically ~10^{11} to 10^{13} molecules cm^{-2}) and antibodies (~10^{10} to 10^{11} molecules cm^{-2}) in electrochemical biosensors.[3–6] Experiments and simulations were performed with a pulse time of 10 ms, a step height of 5 mV and an amplitude of 20 mV, using 3 mm GCE working electrodes. Experimental SWVs used 100 mM phosphate buffer at pH 7 and 25 °C.

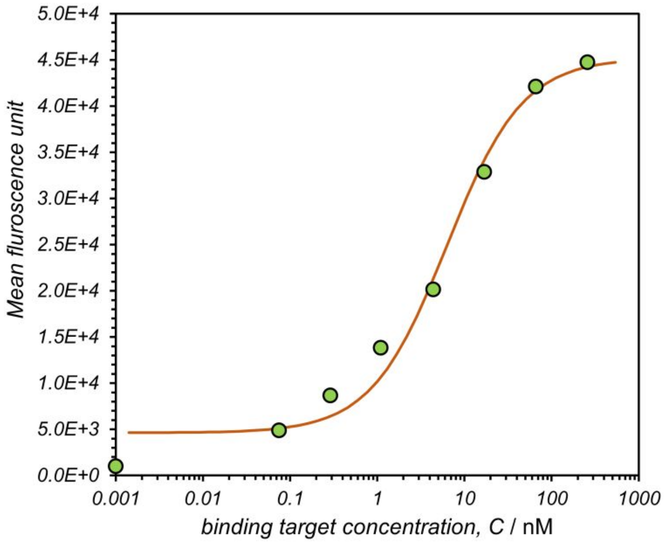

Figure S5. Binding affinity of the fibronectin monobody FL063 to several concentrations of lysozyme was checked through yeast surface display method. The fluorescence measurements of yeast cells expressing FL063 protein on targeting increasing concentrations of lysozyme in solution created a sigmoidal curve with a K_d value of 1.7 nM demonstrating high binding affinity.

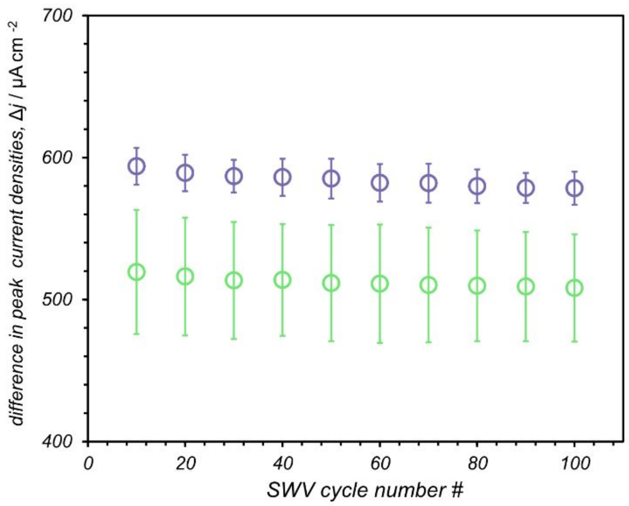

Figure S6. Electrical stability of the biosensor was studied by continuously applying 100 scans of square wave current (pulse width 10ms) in 100 mM PB at room temperature for 90 minutes. No change in current is seen in the monobody immobilized GCE in the presence (green) and absence (purple) of the target lysozyme. Error bars represent one standard deviation from the mean, $n = 3$.

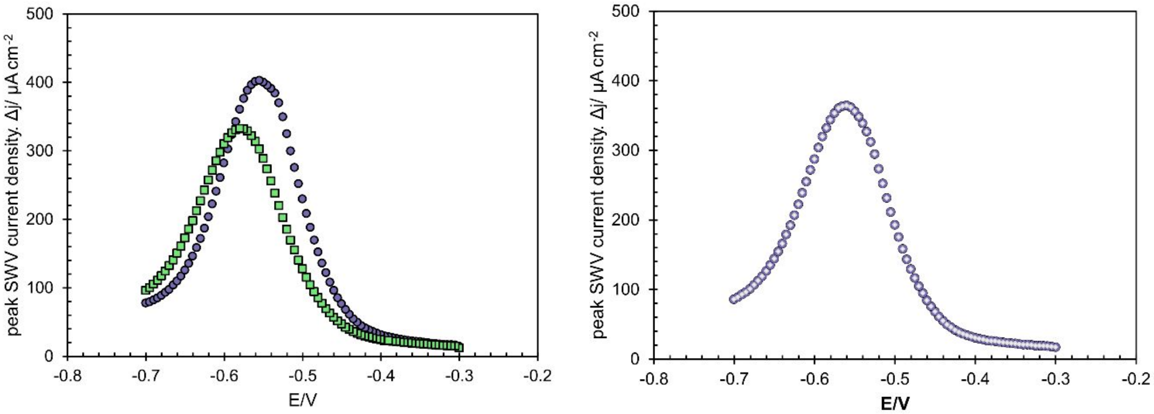

Figure S7. Representative square wave voltammograms of 0.5 mM anthraquinone-2-sulfonate using FL063-modified glassy carbon electrodes (GCEs) before (purple circles) or after incubation in solutions containing either 45 mM lysozyme (left, green squares) or 45 mM BSA (right, white circles). These results, combined with the corresponding $FcNMe_3$ results, demonstrate the compatibility of monobody-based electrochemical biosensors to a variety of redox probes. Experiments were performed using 100 mM phosphate buffer (pH 7.0) at 25 °C with a pulse time of 10 ms, a step height of 5 mV, and an amplitude of 20 mV.

Table S1. Summary of previously reported biosensors for lysozyme in biological media.

Sensor Type	Biorecognition Element	Detection Method	Sample Matrix	Sensitivity (LOD/LOQ)	Ref.	Platform Notes
Monobody-based electrochemical sensor	Engineered monobody	Square wave voltammetry	Biological fluids	LOD 87 nM, LOQ 290 nM	This work	Highly robust, modular, complex-compatible
Electrochemical aptamer–antibody sandwich assay	DNA aptamer & antibody	Differential pulse voltammetry (DPV)	Wine, bio samples	4.3 fM	[7]	Multiplex, high specificity
SPR AuNP-MIP	MIP + AuNP	Surface Plasmon Resonance	Plasma, urine, tears	0.008 µg/mL (~0.56 nM)	[8]	Durable, clinical potential
PEDMAH nanoparticle SPR	PEDMAH nanoparticle	Surface Plasmon Resonance	Egg white	0.084 nM	[8]	Stable in complex samples
FRET Aptasensor	DNA aptamer	FRET fluorescence	Serum	85 nM	[9]	Rapid, sensitive optical
Graphene FET Aptasensor	DNA aptamer	Field Effect Transistor	PBS buffer	10 nM	[10]	Label-free, real-time
Nanoaptasensor (colorimetric)	DNA aptamer	Colorimetry (AuNP aggregate)	Saliva, serum	~0.5 pM	[11]	Ultra-sensitive, naked-eye
MIP@MNP/QD sensor	MIP + magnetic/QD	Fluorescence	Urine, egg white	~4.5 nM	[8]	Stable, multiplexable
DNAzyme electrochemical sensor	DNAzyme	Electrochemistry	Serum, buffer	0.001 nM	[12]	Highly sensitive, immobilization flexible
ELISA / Immunoassay	Antibody	Spectrophotometric (ELISA)	Egg white, plasma	~1 nM	[13]	Gold standard clinical diagnostics

Expression and purification of monobody protein FL063

Chemically competent *E. coli* T7-based expression cells (T7 Express) were transformed with the monobody expression plasmid by heat shock. Briefly, cells were thawed on wet ice, and 0.5 - 5 µL plasmid DNA was added directly to 50 µL cells and mixed by gentle tapping. The mixture was incubated on ice for 30 min, heat-shocked at 37 °C for 90 seconds and returned to ice for 5 min. Room-temperature super optimal broth (SOB) medium (950 µL) was added, and cells were recovered at 37 °C for 1 - 2 h in a shaking incubator at 250 rpm. Cells (200 µL) were spread on Luria broth (LB) agar containing the appropriate antibiotic and incubated inverted overnight at 37 °C. Plates were stored at 4 °C until use.

For expression, a single colony was inoculated into ~5 mL LB supplemented with kanamycin (50 µg mL^{-1}) and grown at 37 °C in a shaking incubator at 250 rpm overnight to obtain a saturated starter culture. The following day, a solution of 1 L LB with kanamycin (50 µg mL^{-1}) was inoculated with 5 mL starter culture and incubated at 37 °C in a shaking incubator at 250 rpm until the culture reached an optical density of $A_{600} \approx 1.0$. Expression was induced by adding 1 mL of 0.5 M isopropyl-β-D-thiogalactoside (IPTG) to each 1 L culture (0.5 mM final IPTG concentration). The shaker temperature was reduced to <20 °C by opening the incubator door for 10 min to cool the culture, and incubation proceeded for 6 h at room temperature in a shaking incubator at 250 rpm.

Cells were harvested by centrifugation at 3,200 × g for 15 min at 4 - 10 °C. Supernatant was discarded and pellets were combined. For cell lysis, pellets were resuspended in 10 mL commercial lysis buffer per liter of culture (commercial lysis buffer contained 50 mM Tris-HCl pH 8.0, 300 mM NaCl, 10 mM imidazole, plus protease inhibitors as suggested by the buffer manufacturer). The suspension was transferred to 15 mL tubes and subjected to four freeze–thaw cycles. Lysates were clarified at 12,000 × g for 10 min at 10 °C, and supernatants were sequentially filtered through 0.45 µm and 0.2 µm syringe filters. Clarified lysates were kept at 4 °C prior to purification.

The protein purification was performed by FPLC on an ÄKTA Pure system using a HisTrap 5 mL Ni^{2+}-sepharose column at 4 °C. The column was equilibrated with 5 - 10 column volumes of equilibration/wash buffer (50 mM Tris-HCl pH 8.0, 300 mM NaCl, and 20 - 30 mM imidazole) at a flow rate of 1.0 mL min^{-1}. Clarified lysate was loaded at 0.5 - 1.0 mL min^{-1}. The column was washed with 10 - 20 column volumes of wash buffer until the 280 nm absorbance, A_{280}, baseline stabilized. Bound monobody was eluted by step elution with elution buffer (50 mM Tris-HCl pH 8.0, 300 mM NaCl, and 250 mM imidazole). Fractions containing the target protein (by A_{280} and SDS-PAGE) were pooled and buffer-exchanged into a storage buffer (20 - 50 mM Tris-HCl pH 7.5 - 8.0, and 150 - 300 mM NaCl) by desalting column. Protein concentration was determined by absorbance at 280 nm, A_{280}, using the sequence-specific extinction coefficient. Purity and expression were assessed by SDS-PAGE.

Table S2. Amino acid sequences of monobodies and DNA sequences of relevant primers used in this study.

FL063:<br><br>MASSSDSPRNLEVTNATPNSLTISWDHYSYAKYYRITYGETGGNSSSQEFTVPGYSSATISGLK PGQDYTITVYAVTTIGYNSNPISINYRTEIDKPSQGSCGSHHHHHH
Fn3103:<br><br>SSDSPRNLEVTNATPNSLTISWDHYSYAKYYRITYGETGGNSPSQEFTVPGYDNATISGLKPGQ DYTITVYAVTDVSYASSPISINYRTEIDKPSQ
Fn3G:<br><br>SSDSPRNLEVTNATPNSLTISWDHYPYAKYYRITYGETGGNSPSQEFTVPGYTNATISGLKPGQ DYTITVYAVTDASYAPNPISINYRTEIDKPSQ
The following primers have been used to amplify the fibronectin clones from the yeast display vector (pCT vector; Addgene plasmid #41843), digest using restriction enzymes (BamHI and NheI enzymes) and ligate into the protein production vector (pET-21 vector):
Forward Primer GeneAmp5 gene amplification: CGACGATTGAAGGTAGATACCCATACG
Reverse Primer GeneAmp3 gene amplification: ATCTCGAGCTATTACAAGTCCTCTTC

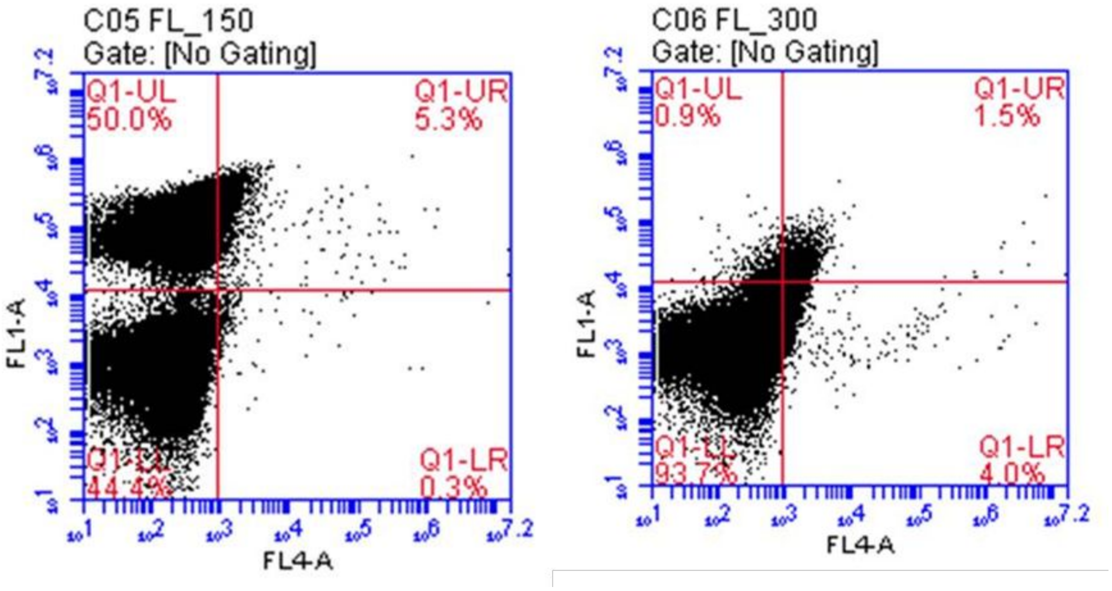

Figure S8. Fluorescence-assisted flow cytometry data showing binding populations of FL063 in the upper right quadrant (double positive) of the plots for 5 nM (left) and 50 nM (right) biotinylated lysozyme. The second quadrant population corresponding to yeast cells expressing the monobody bound to target lysozyme. The biotinylated lysozyme was labeled with Alexa Fluor 488, and yeast cells were stained with Alexa Fluor 647. The double-positive signal in the second quadrant indicates successful binding between the monobody-expressing yeast cells and the target lysozyme.

Table S3. Binding kinetics of FL063 using the mean fluorescence data obtained from flow cytometry.

lysozyme concentration (nM)	median fluorescence signal	Fitted Signal Y_{fit}=A*(F_{min}+(F_{max}-F_{min}) *(S/(S+K_D)))	Residual Error	Residual Error^2
5	61148	62002.02	854.03	729359.5
50	83812	79404.38	-4407.62	19427137.8
150	84668.5	81090.29	-3578.2	12803528.0
500	83481.5	81697.41	-1784.09	3182975.0
750	72823	81784.88	8961.88	80315361.9

F_{min}	F_{max}	K_D	SSR
-6.97588E- 05	81960.39	1.61	1.16E+08

References

(1) Allongue, P.; Delamar, M.; Desbat, B.; Fagebaume, O.; Hitmi, R.; Pinson, J.; Savéant, J.-M. Covalent Modification of Carbon Surfaces by Aryl Radicals Generated from the Electrochemical Reduction of Diazonium Salts. *J Am Chem Soc* **1997**, *119* (1), 201–207. https://doi.org/10.1021/ja963354s.

(2) Polsky, R.; Harper, J. C.; Wheeler, D. R.; Dirk, S. M.; Arango, D. C.; Brozik, S. M. Electrically Addressable Diazonium-Functionalized Antibodies for Multianalyte Electrochemical Sensor Applications. *Biosens Bioelectron* **2008**, *23*, 757–764. https://doi.org/10.1016/j.bios.2007.08.013.

(3) Caroselli, R.; García Castelló, J.; Escorihuela, J.; Bañuls, M.; Maquieira, Á.; García-Rupérez, J. Experimental Study of the Oriented Immobilization of Antibodies on Photonic Sensing Structures by Using Protein A as an Intermediate Layer. *Sensors* **2018**, *18* (4), 1012. https://doi.org/10.3390/s18041012.

(4) Barbosa, A. I.; Edwards, A. D.; Reis, N. M. Antibody Surface Coverage Drives Matrix Interference in Microfluidic Capillary Immunoassays. *ACS Sens* **2021**, *6* (7), 2682–2690. https://doi.org/10.1021/acssensors.1c00704.

(5) Wang, W.-W.; Han, X.; Chu, L.-Q. Polyadenine-Mediated Immobilization of Aptamers on a Gold Substrate for the Direct Detection of Bacterial Pathogens. *Analytical Sciences* **2019**, *35* (9), 967–972. https://doi.org/10.2116/analsci.19P110.

(6) Jeddi, I.; Saiz, L. Computational Design of Single-Stranded DNA Hairpin Aptamers Immobilized on a Biosensor Substrate. *Sci Rep* **2021**, *11* (1), 10984. https://doi.org/10.1038/s41598-021-88796-2.

(7) Ocaña, C.; Hayat, A.; Mishra, R.; Vasilescu, A.; Del Valle, M.; Marty, J. L. A Novel Electrochemical Aptamer–Antibody Sandwich Assay for Lysozyme Detection. *Analyst* **2015**, *140* (12), 4148–4153. https://doi.org/10.1039/C5AN00243E.

(8) Eriş, Ş.; Çimen, D.; Denizli, A. Lysozyme-Imprinted Surface Plasmon Resonance Chips Decorated with Gold Nanoparticles for Lysozyme Detection. *ACS Omega* **2025**, *10* (26), 28055–28064. https://doi.org/10.1021/acsomega.5c01607.

(9) Liu, M.; Zhuang, H.; Zhang, Y.; Jia, Y. A Sandwich FRET Biosensor for Lysozyme Detection Based on Peptide-Functionalized Gold Nanoparticles and FAM-Labeled Aptamer. *Talanta* **2024**, *276*, 126226. https://doi.org/10.1016/J.TALANTA.2024.126226.

(10) Ghosh, S.; Khan, N. I.; Tsavalas, J. G.; Song, E. Selective Detection of Lysozyme Biomarker Utilizing Large Area Chemical Vapor Deposition-Grown Graphene-Based

Field-Effect Transistor. *Front Bioeng Biotechnol* **2018**, *6* (MAR), 352217. https://doi.org/10.3389/fbioe.2018.00029.

(11) Truong, P. L.; Thao, N. T. T.; Le Huyen, H. T.; Nguyen, T. H. Ultrasensitive Detection of Lysozyme upon Conformational Change of DNA Duplex. *J Nanomater* **2022**, *2022* (1), 1107081. https://doi.org/10.1155/2022/1107081.

(12) Khan, S.; Burciu, B.; Filipe, C. D. M.; Li, Y.; Dellinger, K.; Didar, T. F. DNAzyme-Based Biosensors: Immobilization Strategies, Applications, and Future Prospective. *ACS Nano* **2021**, *15* (9), 13943–13969. https://doi.org/10.1021/acsnano.1c04327.

(13) Lee, S.; Hwang, L.; Lee, J. Y.; Yang, T.; Jho, J. Y.; Park, J. H. Multiday Operable Ionic Polymer-Metal Composites Prepared Using a Stacking Method for Practical Actuator Applications. *Sens Actuators B Chem* **2022**, *372*, 132616. https://doi.org/10.1016/J.SNB.2022.132616.
